# Critical thinking in the classroom: the historical method and historical discourse as tools for teaching social studies

**DOI:** 10.3389/fsoc.2025.1526437

**Published:** 2025-04-25

**Authors:** Carmen Burgos-Videla, Marcos Parada-Ulloa, Javiera Martínez-Díaz

**Affiliations:** ^1^Instituto IICSE, Universidad de Atacama, Copiapó, Chile; ^2^Vicerrectoría de Administración y Finanzas, Universidad de Tarapacá, Arica, Chile

**Keywords:** critical thinking, historical thinking, historical discourse, historical method, social sciences

## Abstract

**Introduction:**

This article addresses the pressing need to transform the teaching of History and Social Sciences by moving beyond traditional approaches centered on rote memorization and content reproduction. Instead, it advocates for a pedagogy oriented toward the development of critical and reflective thinking, through the incorporation of the historical method and the construction of historical discourse as central pillars of the educational process. The ultimate aim is to strengthen civic education from a critical and contextually grounded perspective.

**Methods:**

The study adopts a hermeneutic and critical-reflexive approach, based on an exhaustive review of national and international academic literature. The hermeneutic circle is employed as a methodological strategy to analyze how pedagogical practices can reinterpret historical sources, challenge dominant narratives, and foster historical dialogue in the classroom.

**Results:**

The research identifies various pedagogical strategies that operationalize the proposed approach, including structured historical debates, school-based research projects, collaborative analysis of primary and secondary sources, and the integrated use of digital technologies. These strategies are articulated with key categories of historical thinking: contextualization, causality, multicausality, continuity, and change. The study also emphasizes the importance of critical source comparison and the integration of multiple perspectives.

**Discussion and conclusion:**

The findings suggest that adopting a socio-critical approach to the teaching of History can transform the classroom into a space for active reflection, fostering a critical and socially engaged citizenry. The study underscores the need to promote teacher training processes that integrate these methodologies, as well as to encourage future empirical research to assess their impact in diverse educational settings. In sum, it advocates for a historical education that forms critically aware individuals, capable of interpreting the past and acting ethically in the present.

## 1 Introduction

The teaching of History and Social Sciences in the classroom plays a fundamental role in shaping critical, reflective citizens committed to social transformation. In a global context characterized by rapid information circulation and ongoing ideological reinterpretations of historical events (Stearns, 1998), relying solely on traditional pedagogical methods centered on memorization is insufficient. A socio-critical approach combining disciplinary and pedagogical knowledge with techniques promoting deep questioning of power structures (Adorno and Horkheimer, 1944; Bernstein, 1971; Foucault, (1975/1995); Parker and Donnelly, 2014) is necessary.

Various Anglo-Saxon (Barton, 2008), Spanish (Santisteban et al., 2010; Prats, 2011a, 2011b; Prats and Santacana, 2011a; Prats and Santacana, 2011b; Prats and Santacana, 2011c), and Latin American studies (Muñoz, 2005; Vásquez, 2014; Salazar-Jiménez et al., 2015; Muñoz, 2022) demonstrate that pedagogical practices combining disciplinary and didactic knowledge with a socio-critical approach can achieve not only an understanding of the social environment but also the formation of individuals capable of acting against social injustices and inequalities (Aisenberg and Alderoqui, 2007). For example, Muñoz (2005) explicitly proposes applying the hermeneutic circle by integrating the critical analysis of historical sources with students’ contemporary experiences, thus fostering a continuous dialogue between past and present that promotes a critical and engaged understanding of reality.

Therefore, the central problem of this research lies precisely in how to enhance historical and social investigation in the classroom by integrating the historical method, historical thinking, and the construction of historical discourse (Ricoeur, 1990; Muñoz, 2005). The articulation of these elements not only enriches teaching but also equips students with critical competencies necessary to challenge dominant narratives and propose transformative alternatives (Gramsci, 1975). It is suggested that the hermeneutic circle (Gadamer, 1977), in this context, allows students to relate specific parts of history to a broader whole, thereby developing a profound and critical understanding of historical phenomena (Hirsch, 1987) and social action (Weber (1922/2021); Prats and Santacana, 2011a). This approach relies on the dialectical relationship between the parts and the whole of a text, enabling iterative interpretation of phenomena and keeping the analysis open to new understandings (Rennie, 2012; Stenner et al., 2017; Dangal and Joshi, 2020; Alsaigh and Coyne, 2021; Martínez Díaz, 2023).

**Figure 1:**
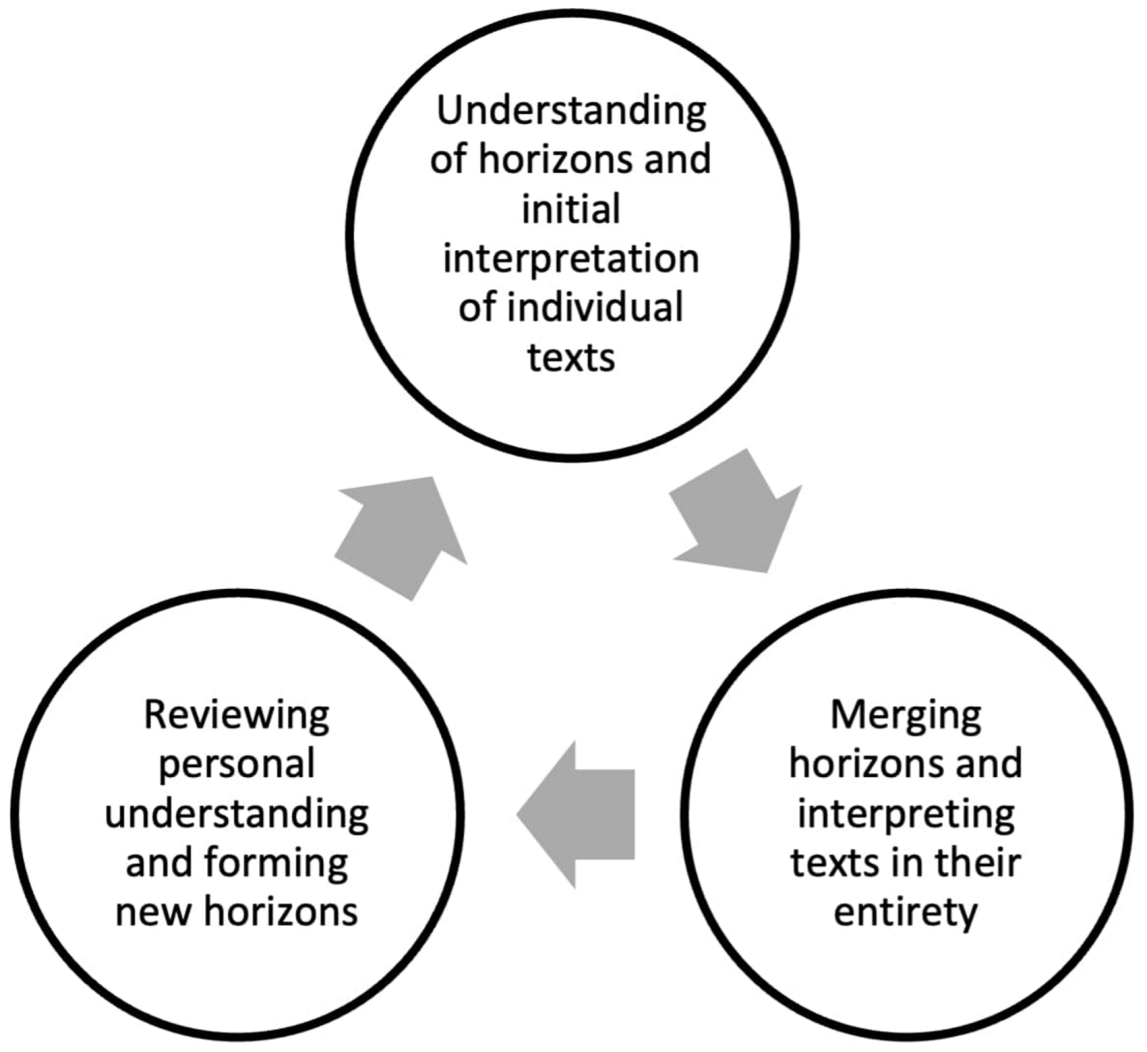
The hermeneutic circle.

This hermeneutic approach does not aim to directly resolve educational problems in history but rather to provide methodological tools that enable questioning and redefining historical narratives (Figure 1). These tools can contribute to critical and flexible learning, with applications in various scientific fields (Andrews, 2021).

Wineburg (2001) suggests that historical thinking involves questioning sources, identifying biases, and recognizing multiple interpretations. Seixas (2000) emphasizes how historical contexts actively connect past social struggles with contemporary challenges, encouraging reflective analysis of the impact of historical structures in the present (Braudel, 1989; Geertz, 1983). This approach, when explicitly applying the hermeneutic circle, enables continuous critical interpretation: students begin with a preliminary understanding of a historical event, analyze diverse sources, question their own preconceptions, and constantly return to the present context, fostering a deep and socially engaged understanding.

Regarding historical discourse, White (1973) argues that historical narratives not only organize past events but also construct meanings imbued with specific interests. In the classroom, VanSledright (2002) and Guha (1982/2002) propose teaching students to deconstruct these narratives, identifying underlying interests and offering inclusive alternatives. Guha (1982/2002), for example, employs the hermeneutic circle by encouraging students to reinterpret colonial history from marginalized voices, integrating initial understandings with emerging critical perspectives, thus facilitating new interpretations that challenge hegemonic narratives.

Lastly, the historical method provides a framework for rigorously investigating and analyzing historical sources. Bloch (1952) emphasizes that the historical method is not limited to the accumulation of data but involves critical reflection on the sources. In the classroom, applying the hermeneutic circle through the historical method entails that students repeatedly return to historical sources after analyzing broader contexts and vice versa, thereby enabling the construction of a critical and informed discourse that addresses current social challenges (Wineburg, 2001).

## 2 Method

This study, given its characteristics, is a documentary investigation. It was developed under a bibliographic design with a critical-reflective approach, focusing on understanding how identifying themes through hermeneutic analysis can be systematically integrated into the teaching of history, promoting the development of historical and critical thinking among students.

The approach was based on the hermeneutic circle applied in two main cycles:

- First Cycle: Key concepts related to historical narratives and their representation in educational contexts were identified (Fernández Palomares, 2003). For instance, by analyzing texts by Prats (2011a, 2011b) and Wineburg (2018), the study explored how historical symbols are used to construct critical discourses in the classroom.
- Second Cycle: The identified concepts were reinterpreted in light of emerging thematic categories, such as the development of critical competencies and the constructivist approach (Stenner et al., 2017). This process established a dynamic and productive relationship between the parts and the whole of the analysis, ensuring that new interpretations enriched the initial categories (Hernández Cardona, 2002; Muñoz, 2013; Santaca, 2005; Pla 2008).

### 2.1 Literature review

As stated by Gómez et al. (2014), the purpose of a bibliographic review is to enable researchers to obtain relevant information on the subject of study. This involves a rigorous search and a proper selection and review of materials to efficiently handle information and achieve the research objectives.

To accomplish this, an exhaustive review of the literature was conducted (Krippendorff, 2019; Alfonzo, 1994; Cué and Oramas, 2008) using databases such as Web of Science (WoS), Scopus, and Scielo, with descriptors like “critical thinking,” “historical thinking,” “historical method,” “hermeneutic circle,” and “History.” These selection descriptors were explicitly defined, considering their pedagogical and theoretical relevance for developing historical and critical thinking, their pedagogical impact, and their contribution to the Social Sciences.

### 2.2 Selection of texts and analysis

A representative sample of texts addressing fundamental issues in history education was constructed.

Selected works include those of Giroux (1990); Prats (2011a, 2011b); Prats and Santacana (2011a, 2011b, 2011c); White (1973); and Wineburg (2018), which delve into critical education and redefine historical narratives from a reflective framework. Additionally, key methodological texts by Bardín (1996), Andréu (2002), and Denzin and Lincoln (2012) provided tools for comparative and content analysis.

The content analysis followed a six-step methodological sequence as outlined by Fernández (2002): (a) identification of analysis topics based on constructs of interest previously established by researchers; (b) data collection using descriptors and selected databases; (c) development of categories and coding schemes using the constructs of interest defined by the researchers, based on the study’s objectives; (d) application of the coding scheme to a small sample of compiled material; (e) coding of the entire corpus by applying the coding scheme to all compiled and preselected documents; and (f) evaluation of coding consistency by verifying the coherence of the processed information. This process involved using a data compilation matrix based on the criteria and coding categories employed, enabling the construction of the text presented.

**Figure 2:**
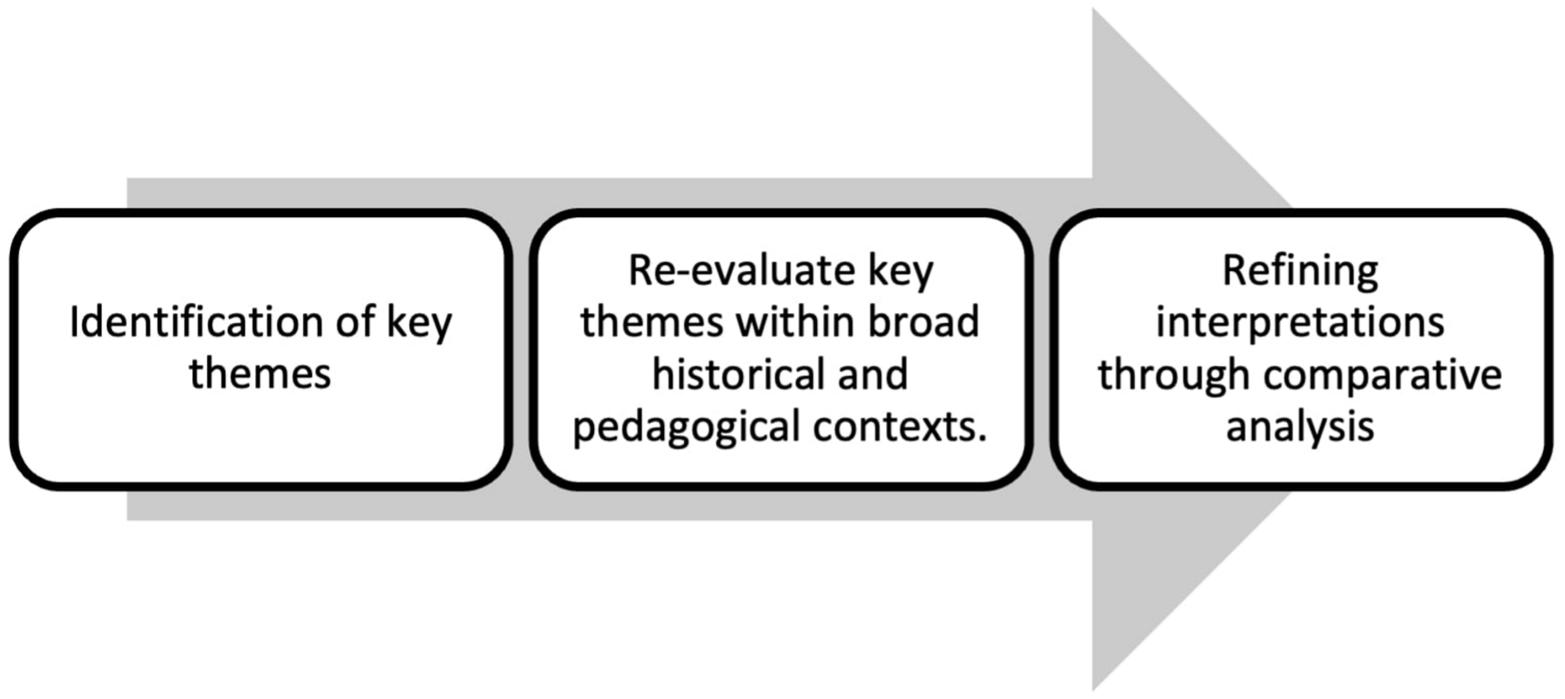
Analytical steps followed.

As a thematic example of the procedure, a set of texts on historical narratives in education was analyzed. This allowed for the identification of emerging categories, which include the development of historical thinking, the promotion of critical competencies, and the reinterpretation of historical narratives in the classroom. The latter is complemented by the hermeneutic analysis presented in Figure 2.

The hermeneutic analysis included an iterative process that followed the steps described in Figure 2:

- The selection of relevant texts through purposive sampling, based on the criteria of pedagogical impact and theoretical relevance.
- A first coding of the texts to identify key concepts related to historical thinking and critical discourse.
- The thematic synthesis of the identified categories, as pedagogical strategies to integrate critical thinking in history teaching.
- The reinterpretation of the results in the light of the hermeneutic theoretical framework, which made it possible to establish a dynamic relationship between the selected texts and the research objectives.

The hermeneutic methodology adopted suggests a perspective for integrating historical and critical thinking into pedagogical practices, fostering a reflective and transformative approach to teaching history.

## 3 Historical thinking

Historical thinking involves a series of fundamental characteristics that guide critical analysis and reflection on history (Wineburg, 2001). In this regard, contextualization—that is, understanding events within their context by considering the social, political, economic, and cultural conditions that influenced them—is essential for profound historical insight (Seixas, 1993; Sandín, 2003). Additionally, causality (Barton, 2008), along with elements of continuity and change, which enable the recognition of both persistent trends and significant historical shifts, facilitates a more comprehensive understanding of the temporal evolution of societies and cultures (Wineburg, 2001).

**Figure 3:**
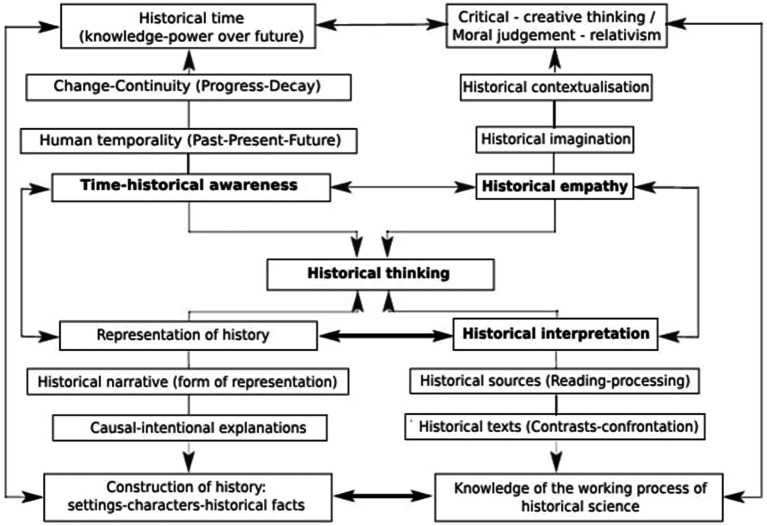
Conceptual model for the development of historical thinking Source: Santisteban et al. (2010, p. 117).

Santisteban et al. (2010) suggest a conceptual model for developing historical thinking, which is based on four fundamental aspects: (a) historical-temporal awareness; (b) representation of history through narration and historical explanation; (c) historical empathy and 155 competencies for contextualization; and (d) interpretation of history based on sources. This model 156 guides the development of research and innovation in the teaching of history (Figure 3).

### 3.1 Application of the hermeneutic circle in history teaching

The application of the hermeneutic circle in pedagogical processes aimed at teaching history would ensure a systematic integration between the critical analysis of historical texts and educational proposals designed for their deep understanding. This methodology allows transcending a static and linear view of history, positioning it as an interpretative, dynamic, and dialectical process that facilitates the connection between past, present, and future. In each section of Table 1, the interpretative steps are explicitly defined, fostering a comprehensive, contextualized, and reflective understanding of the topics addressed from a socio-historical approach.

**Table 1:** Formation of historical thought.

Historical thought				
Conceptual model	Key aspects	Items	Hermeneutic integration	Example
		Historical time		
Historicaltemporal consciousness	Historical awareness, essential in civic education, goes beyond the simple memory of events. It involves a dynamic process of connection between past, present and future, where meanings are constructed to understand the present and anticipate the future. This approach considers time as an interpretive space in which human values and actions are integrated.	Perception of past time as different from the present. Interpretation of time as a flow between change and permanence. orientation of human practice through historical interpretation, Motivation for action through orientation (Santisteban et al., 2010).	This process involves an iterative analysis where historical values are identified in texts and sources. Interpretive steps include: Identify key fragments related to perceptions of time past. Connect these fragments with values of change and permanence identified in the text. Contextualize the interpretations with practical examples to facilitate pedagogical application.	Iterative analysis of texts about historical social movements (such as the labor or student movement) to identify values and actions, connecting them to current social struggles.
	Historical discourse distinguishes between memory and historical	Historical Consciousness	Hermeneutic integration	Example
Representation of history: narration and historical explanation.	Consciousness, although both cover the same field. Memory focuses on historical representations in general culture and highlights the power of the past in pre-rational procedures. It seeks to reveal the various ways of constructing or preserving the past, without considering the structural interrelation between memory and expectation. On the other hand, the discourse on historical consciousness focuses on the rationality of the procedures of historical thought, shaping the past as history. It is especially interested in the forms of representation that give the past that distinctive form. Furthermore, it interprets the impact of history on the future prospects of human life, considering the intentions directed towards the future.	Historical awareness cannot be separated from representation or from the narratives developed by students. It is emphasized that history teaching must achieve that students generate historical, causal and intentional explanations, integrating characters, scenarios and facts in a coherent plot of representation. Narration is essential, but not sufficient to develop historical thinking in adolescents in the school environment.	Regarding the integration of historical representation, students are encouraged to analyze texts that describe historical memory and its influence on culture. This aims to connect these categories to foster critical thinking and analytical skills. Historical awareness is related to linking students’ narratives that allow identifying common patterns and divergent elements; comparing narratives with key historical concepts extracted from pedagogical texts and integrating critical and contextualized narratives.	Critical and analytical account of the coup d’état in Chile (1973), relating historical memory with historical consciousness to underst and its contemporary impact and develop alternative discourses.
	He points out that in the past people not only had different ways of life, but also different experiences,	Teaching and creativity	Hermeneutic integration	Example
Historical empathy and the skills to contextualize	Norms and belief systems. To understand these situations, historians resort to empathic understanding, imagining the past and contextualizing it according to contemporary perceptions and moral judgments. He criticizes empathy as an illusion, arguing that although it requires historical imagination and contextualization, it is essential in the teaching of history. School history contributes to the development of imagination, empathy and moral education. Empathy as a procedural concept helps us understand the motivations of past actors, even if they seem wrong to us now. It is argued that educators should guide students to discover why certain events are considered bad, thus encouraging the use of empathy and the making of moral judgments in history.	He advocates that history teaching should develop critical and creative thinking; criticism allows us to evaluate the past and establish comparisons and relationships with the present. Creativity, on the other hand, enables us to imagine alternative futures in the evolution of current social problems.	Historical empathy allows us to understand past motivations within their own contexts, even if their actions may be questionable today. It is essential to foster moral education and cultural understanding. In this context, the application of hermeneutics would help to analyze historical sources, highlighting norms and beliefs specific to each context; identify normative differences and establish connections with contemporary judgments; and integrate pedagogical strategies that foster empathy and critical moral thinking. History teaching must balance critical thinking and creativity. Criticism analyzes the past to assess the present, while creativity imagines alternative futures. In this sense, the hermeneutic proposal requires identifying how pedagogical texts promote critical and creative skills; categorizing skills according to their application in historical problems and designing activities that reflect this duality, using sources to generate alternative scenarios.	Study of cases such as slavery or colonization, using primary sources to develop historical empathy and understand cultural and moral contexts of the past.
Interpretation of history from sources	According to Ankersmit (2004), when interpreting historical data or sources, we incorporate our historical experience. In teaching history, students’ historical experience can be a motivating and comprehensive tool. In order to develop historical thinking, historical sources become central elements in the educational process. Work with sources should focus on historical problems, where students apply their historical experience to develop historical competence.	Teaching history through historical sources has several benefits:	Hermeneutic integration	Example
		It helps to overcome the structure of textbooks, allows us to learn about recent history and establish connections with other realities, generates historical knowledge as a debatable knowledge, makes it easier for students to delve into the problematic content of the discipline, puts into play the concept of objectivity as opposed to manuals or historiographic texts, allows us to contemplate the past in a natural state, directly involves students with the past, encourages the student’s protagonism and autonomy in historical reconstruction, diversifies the teaching and learning process, and enriches educational experiences.	Interpreting sources allows students to develop historical skills by solving historical problems and reflecting on their current relevance. The elements are related to overcoming the traditional structure of textbooks; knowing recent history and connecting with other realities; generating historical knowledge as debatable knowledge and fostering student autonomy in historical reconstruction. This allows for hermeneutic linkage that includes the selection of historical sources representative of relevant problems; analysis of sources through guiding questions that encourage debate and interpretation; and the design of activities where students reconstruct historical facts, applying criteria of objectivity and discussion.	Using personal letters or historical newspapers to enable students to reconstruct alternative narratives about historical events, facilitating debates about historical objectivity and subjectivity

Sources: Own elaboration.

The hermeneutic circle, as suggested, would not only strengthen interpretative capacities in addressing historical sources but also promote the integration of essential values and competencies among students, such as historical empathy, critical thinking, and creativity. These elements are fundamental in forming reflective and engaged citizens capable of critically dialoguing with their past and projecting alternatives for their future.

From this perspective, the hermeneutic approach is proposed to drive history and social science education beyond merely transmitting knowledge, emphasizing its historical contextualization and the active participation of students in consciously constructing a more just society that reflects on its own historical processes (Berger and Luckmann, 1995).

It is important to note that the claims presented here explicitly restrict the scope of the hermeneutic method to the specific domain of teaching history and social sciences. This avoids undue generalizations that could suggest the universal necessity of this critical methodology for students across all disciplines. Instead, the suggested nature of the approach is highlighted, encouraging its reflective consideration in the educational context without implying its mandatory or extensive application to other fields of knowledge.

## 4 Construction of historical discourse

The construction of historical discourse is not a neutral and objective process but a selective interpretation of the past influenced by multiple cultural, political, and ideological factors (Braudel, 1989; Cárdenas et al., 1991; Carretero et al., 2002). From this perspective, it is suggested to adopt a hermeneutic approach that involves a circular relationship between the understanding of the whole and its parts, known as the hermeneutic circle. This approach enables progress in historical interpretation by connecting specific elements of the text with the entirety of the narrative.

### 4.1 The hermeneutic circle in historical interpretation

Following Hayden White’s (1973) proposal, historical narration requires selecting a narrative framework, choosing relevant events, and organizing information within a specific structure. In this context, the hermeneutic circle aids historians in identifying key themes and systematically connecting them to broader contexts, thereby enriching the analysis of primary and secondary sources. The continuous interaction between research questions, historical data, and theoretical interpretations ensures conclusions are grounded in a rigorous and documented process (Carretero and Voss, 2004).

It is important to note that these statements are presented as methodological suggestions, aiming to consider the hermeneutic circle as a useful tool for constructing historical discourse, without advocating its application as exclusive or universal. The proposal seeks to provide guidelines that enrich investigative practices in the field of history, fostering a critical and contextualized approach.

### 4.2 Pedagogical suggestions for history teaching

From a pedagogical perspective, applying the hermeneutic circle would involve developing educational strategies based on the comparative and iterative analysis of historical sources. Rather than limiting the process to a linear and guided analysis, it is suggested that students contrast various sources and reformulate their interpretations as they acquire new perspectives. For instance, when addressing the colonization of the Americas, comparing Spanish chronicles with Indigenous accounts would reveal substantial differences in the construction of historical discourse.

From a sociological perspective, the hermeneutic methodology could address issues such as cultural hegemony and Eurocentrism in history education (Said, 1978). This approach would promote the identification of privileged narratives and foster the development of a critical and plural understanding of historical processes. Thus, the hermeneutic circle serves as a key tool to encourage a critical pedagogy that questions dominant representations and enhances students’ analytical capacities.

### 4.3 Fostering critical historical thinking

The central objective of historical discourse should be the development of critical and reflective thinking in students, enabling them to analyze the historical nature of events and actively participate in constructing history. As Domínguez (2008) asserts, this would involve both mastering structural historical concepts and understanding the uniqueness of the studied processes.

The hermeneutic approach is suggested to support this objective by systematically connecting identified thematic categories with pedagogical strategies used in the classroom. In educational practice, the hermeneutic circle would guide the interpretative process through clearly defined stages:

- Identification of relevant themes in historical sources.
- Contextualization of these themes within a broader historical framework.
- Synthesis of conclusions that link evidence to fundamental interpretations (Chaux et al., 2004; Reisman, 2012).

This methodology would help ensure that pedagogical recommendations are directly derived from a rigorous and systematic analysis, fostering essential skills in students, such as identifying biases, comparing perspectives, and constructing evidence-based arguments (Prats, 2011a, 2011b).

### 4.4 Critical reflection and the formation of historical identity

Historical discourse influences the formation of students’ identity and historical awareness (Domínguez, 2008). To avoid biased or oversimplified interpretations, it is suggested that the hermeneutic circle clarifies the selection of key themes, aiming to adequately address the specific demands of the discipline (Carretero and Voss, 2004). Donovan and Brasford (2005) emphasize the importance of designing educational materials that reflect a deep understanding of historical processes, promoting in students a critical and pluralistic view of the past.

The construction of historical discourse should be understood as an active and reflective process involving both historians and educators. In this context, the hermeneutic circle would ensure a clear and systematic connection between the identified themes and the analysis conducted, thereby fostering a more rigorous and meaningful understanding of the past.

However, it is essential to note that the reflections presented here are confined to the domain of history and social sciences. The proposal does not aim to advocate the hermeneutic approach as an obligatory or universal method for all disciplines, thus avoiding generalizations that could misinterpret the intended pedagogical purpose. The aim is to offer reflective guidelines that can enrich educational practices in specific contexts of historical and social education (Table 2).

**Table 2:** Historical thought, discourse and method and teaching strategies.

Concepts	Teaching strategies	Theoretical explanation	Hermeneutical approach
Historical thought	Use of primary sources, critical analysis, contextualization of events, debates	Historical thinking involves analyzing, interpreting, and understanding historical events by considering their context and causal relationships. It fosters the development of critical thinking and allows us to recognize continuities and changes in history.	Using the hermeneutic circle approach, the initial understanding of individual events is connected to the interpretation of broader patterns, thus allowing for in-depth reflection on continuities and changes in history. This approach fosters the development of critical thinking by systematically integrating primary sources with existing historiographical narratives.
Historical speeches	Creation of historical narratives, case analysis, discussion of different perspectives	Historical discourse is constructed through a process of selecting, interpreting and organizing events to communicate a coherent narrative.	The hermeneutic circle establishes a continuous relationship between the questions posed, the evidence and the insights gained from historical narratives. For example, in case analysis, key themes are identified through critical evaluation of sources and their contextualization, linking contrasting perspectives to a grounded interpretation. This process ensures that the resulting narratives reflect a rigorous and systematic analysis.
Historical method	Rigorous research, analysis and evaluation of sources, application of the comparative method	The historical method is a tool for researching and analyzing the past with academic rigor. It involves evaluating primary and secondary sources, formulating questions, and using comparative methods to interpret historical facts.	Within the framework of the hermeneutic circle, this method involves an iterative process where primary and secondary sources are examined, formulating questions that guide the interpretation of the data. The thematic category emerges by relating specific findings to broader patterns. For example, when applying the comparative method, different evidence is contrasted to identify continuities and ruptures, ensuring that conclusions are derived from a systematic and well-founded analysis.

Source: Own elaboration.

The integration of the historical method into classroom practices is grounded in a systematic process combining hermeneutic analysis with didactic strategies based on primary and secondary sources. This approach enables students to analyze history from diverse perspectives, using historical documents, oral testimonies, and cultural artifacts. The hermeneutic circle suggests an iterative relationship between the initial understanding of events and their reinterpretation within broader contexts. For example, comparative analysis of secondary sources would not only enrich historical discourse but also connect diverse perspectives with the key interpretations generated.

### 4.5 Developing critical thinking and using digital technologies

Developing critical thinking recommends implementing historical debates in which students discuss different interpretations of historical events and their contemporary relevance (Fallace and Neem, 2005; Wilke et al., 2022). Through the hermeneutic circle, these debates could be linked to previously analyzed evidence, fostering deep critical understanding. This process would promote not only the ability to argue based on evidence but also the use of digital tools, such as multimedia archives and simulations, to enhance students’ engagement with the past.

Digital technologies, such as virtual reality, can allow students to actively experience history, thus reinforcing the connection between individual interpretations and broader historical contexts (Jonassen et al., 2003; Wineburg, 2018). The pedagogical use of these tools is suggested to promote experiential understanding that complements theoretical analysis.

### 4.6 Didactic strategies for critical analysis

Developing didactic strategies that promote the analysis of primary sources, discussion of different historical perspectives, and the fostering of critical thinking is essential (Bell, 2010). This approach proposes addressing sensitive and controversial topics in history, such as conflicts, human rights violations, and social inequalities, through ethical and respectful analysis (Domínguez, 2008). These suggestions aim to provide methodological guidelines that strengthen students’ critical and reflective capacities, without intending rigid or universal application across all educational contexts.

### 4.7 Process of constructing historical discourse

The process of constructing historical discourse can be divided into several stages, each closely linked to the hermeneutic circle to ensure deep and rigorous understanding of history:

1. Selection and analysis of historical sources: According to Perafán Cabrera (2013), primary and secondary sources provide different levels of direct information about the past, while secondary sources offer interpretations. The hermeneutic circle enables the connection between them, extracting key themes through an iterative process of understanding and reinterpretation (Wineburg, 2001).
2. Critical interpretation of information: Once the sources are selected, it is recommended that students interpret and analyze historical information critically, contextualizing, interpreting, and assessing the reliability and relevance of the sources used. The hermeneutic circle at this level helps connect initial interpretations to a broader and deeper understanding of the historical context. Carretero et al. (2013) highlight that this methodology fosters the ability to contrast different historical interpretations, linking individual perspectives with diverse historical patterns.
3. Organization and structuring of historical discourse: At this stage, students are encouraged to organize historical information coherently, selecting relevant events and establishing logical connections between them. Yilmaz (2008) emphasizes that coherent structuring is fundamental for constructing well-founded and solid arguments.
4. Constructing evidence-based arguments: Students are recommended to synthesize the collected information to develop evidence-based historical arguments. According to Wineburg (2001), this process fosters complex analytical skills, promoting the ability to relate employed methodologies with the construction of historical discourse.

### 4.8 Integrating the historical method in the classroom

The integration of the historical method in the classroom through the hermeneutic approach suggests an educational practice that transcends the mere accumulation of knowledge, focusing instead on critical analysis, contextualization, and historical reflection. However, it is important to emphasize that these proposals are presented as methodological suggestions that could enrich teaching practices, without implying normative or exclusive application across all educational contexts. The goal is to generate pedagogical reflections that foster critical thinking and deep analysis of historical processes.

## 5 Historical method

The historical method constitutes a valuable tool for developing critical and analytical thinking in students, as it fosters a deeper understanding of the past. Wineburg (2001) highlights that the application of the historical method not only enables students to acquire research, interpretation, and analysis skills in relation to historical sources but also confronts them with the cognitive challenges inherent to history as a discipline. These statements are presented as methodological suggestions aimed at enriching history education through the use of the historical method, without intending to establish its normative or exclusive application across all educational contexts.

### 5.1 The historical method: a critical and reflective perspective

This perspective is complemented by the contributions of authors such as Carr (1966); Ariès and Duby (1987-1989); Burke (2003); and Le Goff (2005), who emphasize the importance of a systematic, critical, and reflective process for achieving historically grounded knowledge. In this context, the historical method should be understood as a cognitive and reflective process that requires systematic and critical approaches to achieve well-founded knowledge with a high level of certainty (Benejam et al., 2002). For example, considering current historiographical approaches—historical-critical, biographical, comparative, microhistorical, structural-systemic, socio-historical, economic, and anthropological (Duby and Guy, 1996; Benejam et al., 2002)—it is suggested to promote dialogue between the past and the present to gain a deep understanding of historical phenomena. This approach would enable students to establish relationships between different periods and contexts, fostering broad and contextualized analysis.

### 5.2 Suggestions for using the historical method in the classroom

It is proposed to complement the historical method with the hermeneutic circle to enrich investigative practices in history. This methodological approach would allow students to develop critical and analytical skills by making the interpretative process visible and systematic, connecting historical sources with their critical analysis. In the educational sphere, methods such as expository teaching, discovery learning, inductive approaches, analysis of continuity and change, as well as disciplinary research (Pereyra, 1995; Santisteban et al., 2010; Almansa, 2018) are suggested. These strategies can facilitate students’ engagement with history not only through the acquisition of factual knowledge but also through the development of analytical skills that enable them to understand the complexity of the past and its implications in the present.

### 5.3 Integrating methods and approaches: a reflective proposal

The integration of these methods and approaches suggests that the teaching and learning process of history should go beyond mere content transmission and instead promote the development of critical and reflective competencies. This pedagogical approach encourages students to question, analyze, and interpret historical events from multiple perspectives, taking into account both the context of the sources’ production and contemporary interpretations.

## 6 The classroom: a historical laboratory

The classroom, conceived as a historical laboratory, allows transforming the teaching of History and.

Social Sciences into a dynamic space of active research and critical dialogue (Bloch, 1952; Giroux, 1990; Prats, 2011a, 2011b). This socio-critical approach not only rejects the unidirectional transmission of knowledge, but also places students as protagonists in the construction of historical knowledge, developing critical, social and analytical skills that transcend the memorization of information. Giroux (1990) and Apple (1997) emphasize that learning should be oriented towards the analysis of social structures, the evaluation of dominant historical narratives, and the exploration of alternative perspectives.

From a socio-critical perspective, the hermeneutic circle and thematic categorization become essential tools to systematically connect methodological processes, allowing students to develop a critical understanding of historical narratives. It is suggested that students analyze primary and secondary sources to achieve a deep understanding of historical events and their impact on society. Rather than merely memorizing dates and names, they would be encouraged to question dominant narratives, explore different perspectives, and analyze power dynamics present in history (Salazar-Jiménez et al., 2015). Through the critical analysis of historical sources, students would learn to evaluate the accuracy and objectivity of dominant historical narratives. This entails considering biases and omissions within sources, as well as analyzing the various perspectives and voices present in history.

To develop these critical and social skills, it is important to foster dialogue and debate in the classroom. Students should have the opportunity to express their opinions, listen to their peers’ perspectives, and construct arguments grounded in historical evidence. By participating in reflective discussions and exchanging ideas, students learn to communicate effectively and to respect others’ viewpoints (Maggioni et al., 2009).

Through the critical analysis of historical sources, students learn to evaluate the veracity and objectivity of dominant historical narratives. This involves considering the biases and omissions present in the sources, as well as analyzing the different perspectives and voices present in history.

To develop these critical and social skills, it is important to encourage dialogue and debate in the classroom. Students should have the opportunity to express their opinions, listen to the perspectives of their peers, and construct arguments grounded in historical evidence. By engaging in thoughtful discussions and the exchange of ideas, students learn to communicate effectively and respect the opinions of others (Maggioni et al., 2009).

Linking the socio-critical approach to the methodological process would help organize knowledge around axes that allow students to connect past events with current problems. This methodology fosters meaningful learning by facilitating the classification, analysis and synthesis of information, fundamental elements for the construction of a coherent and well-founded historical discourse. In this way, an explicit link would be achieved between the methodological process and the pedagogical results, reinforcing the students’ capacity to generate their own historical narratives.

1. Historical discourse construction: students participating in this type of methodology would develop advanced skills to interpret and construct historical discourses that integrate multiple perspectives, critically evaluating dominant narratives. This implies, as Maggioni et al. (2009) point out, learning effective communication skills and the ability to construct grounded arguments.
2. Analysis of social structures: Critical analysis of historical sources allows students to identify the power relations and inequalities that have shaped both the past and the present. This not only enriches their historical understanding, but also promotes greater social and political awareness.
3. Fostering critical dialogue: Reflective classroom discussions foster collaborative learning where different points of view are valued, contributing to the development of communication skills, empathy and respect for the diversity of opinions (Giroux, 1990; Smith and Breakstone, 2015).

In addition, historical research projects could be implemented, where teachers guide students in the analysis and interpretation of historical sources, using strategies such as the formulation of hypotheses, thematic categorization and the hermeneutic circle; encourage active learning, to promote the active participation of students through discussions, group work and the use of digital tools that facilitate access and analysis of sources; strengthen the research skills of teachers, allowing them to carry out rigorous research, master the use of various sources and apply methodologies that enrich pedagogical practice.

## 7 Discussion, relevance, and implications

The integration of the historical method in the classroom can be based on a systematic process that combines hermeneutic analysis with didactic strategies centered on the critical and reflective use of primary and secondary sources. This approach suggests that students explore diverse historical perspectives through original documents, oral testimonies, and cultural artifacts. The hermeneutic circle proposes an iterative relationship between initial understanding and increasingly deep and contextualized interpretation of historical events (Brookfield, 2012). Comparative analysis of secondary sources could not only enrich the construction of historical discourse but also clearly articulate different perspectives with essential identified interpretations.

### 7.1 Historical thinking: beyond a linear narrative

Developing critical thinking may recommend the implementation of historical debates where students have the opportunity to discuss and contrast diverse interpretations of historical events and their contemporary relevance (Fallace and Neem, 2005; Wilke et al., 2022). The hermeneutic circle could strengthen these debates through the systematic use of previously analyzed historical evidence, fostering a structured, well-founded critical understanding. Similarly, the inclusion of digital tools such as multimedia archives and simulations can facilitate active and meaningful student engagement with the past. Innovative technologies, such as virtual reality, suggest that students experience history immersively, consolidating the connection between their individual interpretations and broader historical contexts (Jonassen et al., 2003; Wineburg, 2018).

From a pedagogical perspective, it is advisable to implement didactic strategies that promote rigorous and critical analysis of primary sources as well as open discussion of diverse historical perspectives. This approach can help address sensitive and controversial topics, such as historical conflicts, human rights violations, or social inequalities, through ethical, critical, and respectful analysis (Domínguez, 2008).

### 7.2 The process of constructing historical discourse

The process of constructing historical discourse can be divided into various stages systematically connected to the hermeneutic circle, thus ensuring a comprehensive analysis of the past. The first stage could involve the selection and critical analysis of historical sources. Perafán Cabrera (2013) states that primary sources provide direct and specific information about the past, while secondary sources offer more general interpretations. The hermeneutic circle is suggested as a tool for the critical interconnection of these sources to extract relevant themes through an iterative process of analysis, interpretation, and reinterpretation (Wineburg, 2001).

The second stage corresponds to the deep and contextualized interpretation of the selected historical information. At this stage, students can contextualize, critically evaluate, and contrast the sources to determine their reliability and historical relevance. Carretero et al. (2013) highlight how this hermeneutic method can promote the ability to contrast multiple historical interpretations, integrating individual perspectives into broader, diverse interpretative patterns.

The systematic organization and structuring of historical information can lead to a logical and wellfounded synthesis of historical discourse. Wineburg (2001) argues that this process could enhance the ability to synthesize and construct solid arguments, supported by clearly defined evidence and an explicit critical methodology. In this way, the hermeneutic circle contributes not only to strengthening historical interpretation but also to promoting the comprehensive development of students’ historical and critical thinking.

Tables 3 and 4 are presented summarizing the main findings and their impact on the development of students’ cognitive and critical competencies in the classroom.

**Table 3:** Elements of historical thinking and their impact on students.

Element	Definition	Impact on students
Contextualization	Understanding historical events within their social, political and economic context.	It facilitates students’ ability to analyze events in their complexity.
Causality	Identification of the causes and consequences of historical events.	Improves understanding of the interconnection between historical facts and processes.
Historical empathy	Ability to understand the experiences and beliefs of people from the past.	It encourages critical reflection on cultural and social differences over time.
Change and continuity	Recognition of transformations and continuities in history.	Allows students to identify trends and assess historical impact.

Source: Own elaboration.

**Table 4:** Proposed didactic strategies for the development of historical thinking.

Strategies	Description	Expected results
Use of primary sources	Critical analysis of documents, testimonies and artifacts from the past.	Develops students’ critical and autonomous capacity.
Debate and critical dialogue	Discussion on different interpretations of historical events.	Promotes critical thinking and the ability to formulate reasoned arguments.
Creating historical narratives	Students construct narratives based on analysis of sources.	It encourages creativity and the construction of alternative historical discourses.
Comparative analysis	Comparison between different historical sources to identify biases.	Increases students’ ability to recognize subjectivities in history.

Source: Own elaboration.

In the classroom, this approach can be translated into concrete didactic strategies, such as the possible comparison of primary and secondary sources on the same historical event, the suggested implementation of structured debates presenting different perspectives on a historical process, and the recommended analysis of how certain groups have been marginalized in official accounts. This approach could foster students’ critical analysis skills and strengthen their argumentative competence.

Historical thinking is more than a cognitive skill; it can be conceived as a transformative tool that allows students to analyze historical events from a pluralistic and non-linear perspective. This approach could break away from traditional pedagogy, where history is presented as a chronological sequence of disconnected events, and instead position it as a dynamic process linking the past and the present. Wineburg (2001) and Seixas (2000) emphasize the need to question dominant narratives and build deep understandings through contextualization, causality, and historical empathy. The findings of this study reinforce these ideas by suggesting that students actively engaged in guided analyses not only acquire factual knowledge but also develop critical skills to interpret the complexity of historical relationships. This process fosters the ability to connect seemingly disparate events, bridging different points in the past with contemporary issues. However, it is crucial to highlight a gap in the existing literature: the limited focus on educational contexts with scarce resources or high cultural diversity. This study proposes specific strategies, such as comparative analysis of sources and group work, which can be adapted to these settings, leveraging collaborative dynamics to enrich the construction of historical thinking.

### 7.3 The hermeneutic circle: dynamic and reflective interpretation

The hermeneutic circle, rooted in the theory of Gadamer’s (1977), allows for a constant interaction between the parts and the whole in historical analysis. This approach could prove especially useful in teaching, where students can integrate specific evidence within a broader interpretative framework. Applying this technique fosters critical understanding of historical sources, emphasizing the importance of considering their biases, omissions, and production contexts.

A key contribution of the hermeneutic circle lies in its ability to foster critical and analytical competencies in students. The systematic use of primary and secondary sources facilitates not only the construction of fundamental historical narratives but also enables students to contrast diverse historical perspectives, thereby strengthening their critical and analytical abilities.

From a pedagogical perspective, the hermeneutic circle could enable an interactive and reflective approach that supports meaningful learning. For instance, in an activity on the French Revolution, students could compare primary sources (documents contemporary to the events) with later secondary interpretations, identifying how interpretations vary according to ideological and cultural contexts. In this framework, they could be encouraged to formulate their own interpretations and debate the relevance of these historical events in the present, establishing connections between past and present.

Similarly, when studying specific historical events such as the Cold War, comparative analysis of sources can allow students to recognize underlying ideological and contextual patterns in historical discourse. This is achieved through an iterative process of analysis and interpretation characteristic of the hermeneutic circle, promoting a deep and critical understanding of ideological and narrative constructions in historical texts.

However, the effective application of the hermeneutic circle may face practical challenges, among which is the lack of teacher training in interpretative methodologies. Therefore, this study suggests that initial and ongoing teacher education programs explicitly incorporate the learning and practice of the hermeneutic circle. This could ensure that teachers possess the necessary competencies to apply interpretative methodologies in the classroom, thus promoting a critical, interactive, and reflective approach to teaching history.

### 7.4 Work with sources: the foundation of historical learning

The integration of the historical method in the classroom can be based on a systematic process that combines hermeneutic analysis with didactic strategies focused on primary and secondary sources. This approach proposes enabling students to approach history from multiple perspectives through historical documents, oral testimonies, and specific cultural artifacts such as letters, personal diaries, photographs, or historical objects. Using the hermeneutic circle, it is suggested to establish an iterative process where initial interpretations can be continuously revised in light of a broader and more complex context. For example, while studying the French Revolution, students could analyze primary documents such as speeches by revolutionary leaders alongside contemporary secondary interpretations, identifying similarities and differences that enrich their historical understanding.

Comparative analysis of primary and secondary sources could allow for a deeper exploration of historical discourse while connecting diverse perspectives with fundamental and reflective interpretations. For instance, when addressing the Cold War, comparing official accounts from the United States and the Soviet Union with testimonies from ordinary citizens could provide a broader view of the political and social tensions of the period.

The development of critical thinking may recommend the implementation of structured historical debates, where students confront different interpretations of historical events and explore their contemporary relevance (Fallace and Neem, 2005; Wilke et al., 2022). These debates, strengthened by the hermeneutic circle, could link previously analyzed evidence with wellfounded arguments, promoting a deep and critical understanding. Additionally, using digital tools, such as multimedia archives or interactive simulations, can significantly enhance engagement with the past. Technologies like virtual reality, for instance, could allow students to virtually experience key historical moments, such as virtual visits to ancient Egypt or simulations of the Normandy landings (Jonassen et al., 2003; Wineburg, 2018).

Addressing sensitive historical topics such as armed conflicts, human rights violations, and social inequalities recommends ethical and respectful analysis. For example, critical analysis of sources on Latin American dictatorships could enable students to recognize dominant narratives and question them from diverse ethical and social perspectives (Domínguez, 2008).

The process of constructing historical discourse can be developed across several stages closely linked to the hermeneutic circle, ensuring integral and rigorous interpretation of the past. The first stage could involve the careful selection and critical analysis of available historical sources. Perafán Cabrera (2013) emphasizes the importance of clearly distinguishing between primary and secondary sources and how the hermeneutic circle can extract key themes and significant contextual relationships (Wineburg, 2001).

The second stage consists of the deep interpretation of selected historical information, where students can contextualize historical events and critically evaluate the reliability and potential biases present in the sources. For instance, in analyzing European imperialism in Africa, students could identify recurring historical patterns and critically analyze colonial discourses from diverse cultural and economic perspectives (Carretero et al., 2013).

The stage of organizing and structuring historical information could involve constructing coherent and solid narratives. For example, when studying social movements such as civil rights in the United States, students can develop historical arguments that integrate oral testimonies, government documents, and academic analyses, thereby strengthening their analytical and argumentative competencies (Wineburg, 2001).

The systematic integration of historical thinking, enriched by the hermeneutic circle and interdisciplinarity, can transform history teaching and foster the comprehensive development of critical thinking across various areas of knowledge, thereby nurturing reflective and active citizens.

### 7.5 Sociological analysis to contextualize the importance of critical thinking in civic education

Sociological analysis to contextualize the importance of critical thinking in civic education can be considered a fundamental element. Bourdieu (1991) argues that access to critical tools for historical analysis could not only enable understanding of social structures but also challenge the mechanisms of power reproduction. In this sense, the hermeneutic circle is suggested as a strategy to facilitate the interpretation of the past and generate awareness about the structural conditions shaping contemporary reality.

Freire (1970) complements this idea by proposing that critical pedagogy could serve as a means to foster social agency. Through dialogue and critical reflection, students could analyze historical processes and develop the ability to transform them. For example, in the classroom, students could debate the French Revolution and how its ideals influenced subsequent social movements, thus fostering a deep and critical understanding of historical change.

In this vein, Giroux (1983) and Apple (1997) recommend considering education as a space of resistance, where individuals can develop as critical citizens capable of informed societal intervention. In practice, this could be translated into school projects analyzing and questioning socioeconomic inequality in different contexts, such as housing policies in urban and rural areas.

From a structural perspective, Giddens (1991) emphasizes that historical knowledge could contribute to social reflexivity, enabling individuals to understand their position within the social system and act accordingly. In the classroom, this could be implemented through methodologies that challenge dominant narratives and foster independent thinking. For example, students might contrast political and economic discourses about the 2008 financial crisis to analyze how different actors interpreted the situation according to their interests, thereby developing a critical perspective on the consequences of such interpretations.

The integration of the hermeneutic circle with a sociological approach can enhance the relevance of this study by offering a methodology that could promote critical thinking in history and contribute to the formation of citizens capable of analyzing and transforming social reality.

## 8 Conclusion

The study highlights the potential to transform the teaching of History and Social Sciences into a process that fosters critical, historical, and reflective thinking. The proposal could be expanded by incorporating critical realism as a fundamental epistemological framework (Bhaskar, 1989). This approach is suggested as a way to link hermeneutics with a structural and transformative dimension, strengthening history education as a practice oriented toward social emancipation. In this way, the analysis of historical sources could transcend mere textual interpretation and become a tool for transforming social realities, grounded in the principles of critical pedagogy proposed by Freire (1970).

Far from rote memorization, the proposed approach recommends transforming the classroom into a dynamic laboratory where students, through the use of the historical method, critical analysis of sources, and the construction of historical discourse, become key actors in reinterpreting the past and creating narratives that address contemporary challenges.

The discussion could be deepened through critical analysis that questions the power structures implicit in dominant historical thinking. This approach suggests that historical discourse can function both as a mechanism of ideological reproduction and as a tool for student resistance and agency. It is advisable to integrate theories of social reproduction (Bourdieu and Passeron, 1977) and cultural resistance (Apple, 1997; Giroux, 1983), providing a complex view of the role of history teaching in diverse educational contexts. A concrete example would be a comparative study of the teaching of colonial processes from both subaltern and dominant perspectives, highlighting how historical education can perpetuate or challenge hegemonic discourses.

From a pedagogical perspective, this model could represent a paradigmatic shift, promoting an education focused on critical competencies and the development of analytical skills. Historical thinking can facilitate an understanding of history as a dynamic process and prepare students to question hegemonic narratives and construct sociohistorical alternatives. The integration of the hermeneutic circle is proposed as a strategy not only to rigorously interpret sources but also to systematically integrate evidence with educational objectives, offering a framework for contextualization and critical dialogue.

The findings could emphasize the transformative potential of an education that links the past with contemporary social struggles. By fostering historical empathy and critical awareness, students can become conscious and committed citizens advocating for social justice. This approach could also have implications for educational policy, especially in contexts of high cultural diversity or limited resources, where collaborative strategies and the use of emerging technologies may be essential in overcoming existing barriers.

In the realm of future research, the study proposes exploring the empirical validation of the proposed strategies. Case studies and longitudinal designs are suggested to measure the impact of these approaches on students’ critical thinking and historical empathy. Additionally, it could recommend exploring interdisciplinary approaches that integrate history with other fields, such as sociology, literature, and political science, thereby enriching the learning experience and broadening the scope of developed competencies.

The teaching of History and Social Sciences can be reimagined as a tool to empower students, not only in their understanding of the past but also in their ability to envision and construct equitable futures. This pedagogical model could redefine the role of the classroom and the educator, offering a path toward critical, inclusive, and transformative education.

## 9 Limitations and recommendations

In any study it is important to recognize the limitations that arise when presenting the results. In our case, the main limitation of this study is its theoretical approach, so there is a lack of empirical testing of the recommendations provided in a classroom and thus validate the proposal in greater depth. It is recommended that case studies or mixed methods be used for this purpose. Future research could apply the proposals to measure the actual impact of strategies for developing critical thinking in students and historical empathy. Longitudinal studies could also be conducted to study the application of this type of models in a sustained manner and their impact on the development of these skills in students. It is suggested that the strategies to be implemented should be in contexts with diverse demographic composition. Additionally, it has a dependence on interpretive methods that are always subject to the subjectivity of the interpreter, despite every effort to maintain an objective approach. It is important to remember that a hermeneutic approach may reduce the replicability of the study in some contexts.

## Data Availability

The raw data supporting the conclusions of this article will be made available by the authors, without undue reservation.
